# Superoxide Generation and Its Involvement in the Growth of *Mycobacterium smegmatis*

**DOI:** 10.3389/fmicb.2017.00105

**Published:** 2017-01-30

**Authors:** Amar M. Yeware, Ketaki D. Shurpali, Meghana C. Athalye, Dhiman Sarkar

**Affiliations:** Combi Chem-Bio Resource Center, Organic Chemistry Division, CSIR-National Chemical LaboratoryPune, India

**Keywords:** Reactive oxygen species, superoxide, diphenyleneiodonium chloride, *Mycobacterium smegmatis*, mycobacteria growth, NADH oxidase

## Abstract

Superoxide generation is inevitable in aerobic organisms, most of which have developed mechanisms to detoxify superoxides. However, its significance has not been clearly understood in mycobacteria. This study demonstrates that NADH oxidase is the major source of superoxide in *Mycobacterium smegmatis* and elucidates the involvement of superoxide in *M. smegmatis* growth. The maximum inhibition of superoxide generation was observed in the presence of diphenyleneiodonium chloride (DPI), an NADH oxidase inhibitor, compared to other standard inhibitors. After incubation for 24 h, the number of colony forming units (CFUs) was reduced by 6.8 log_10_ compared to the untreated culture. The inhibitory effect of DPI on *M. smegmatis* was reversed when the same culture was supplemented with menadione and pyrogallol, which are superoxide generators. Thus, this study reports the source of superoxide generation and its involvement in the growth of *M. smegmatis*.

## Introduction

The survival of *Mycobacterium tuberculosis* (Mtb) the causative agent of tuberculosis inside the phagosomes of macrophages is critical for its virulence (Russell, 2001; Schnappinger et al., 2003; Rohde et al., 2007). Reactive oxygen species (ROS) and reactive nitrogen species (RNS) are secreted to kill the foreign bacteria inside macrophages (Chan et al., 1992; Adams et al., 1997; Oberley-Deegan et al., 2010). Indeed, the influence of O_2_ on the growth of *Mycobacterium* spp. is well-known (Wayne and Hayes, 1996; Kumar et al., 2008; Taneja et al., 2010). However, Mtb has evolved protective detoxification mechanisms in response to the exogenous oxidative stress encountered inside the host phagocytes.

ROS includes superoxide radicals, hydrogen peroxide, and hydroxyl radicals (Finkel, 2011). Mycobacterial antioxidant enzymes are known to play an important role in the defense against oxidative stress in macrophages; however, their expression in axenic cultures remains unclear. Mtb encounters ROS in the host and overcomes the oxidative stress through multiple thioredoxin systems that function as the antioxidant defense, such as thioredoxin reductase, thioredoxin C, and TPx (Jaeger et al., 2004). These findings also support that Mtb possesses a thiol-oxidoreductase system along with a superoxide-detoxifying enzyme (SodA) and an integral membrane protein (DoxX) called the membrane-associated oxidoreductase complex (MRC; Nambi et al., 2015). Paradoxically, earlier reports have suggested that a more oxidizing environment leads to the enhanced growth of *M. abscessus* as well as Mtb inside macrophages and a reducing environment inhibits their growth (Meylan et al., 1992; Oberley-Deegan et al., 2010).

**FIGURE 1:**
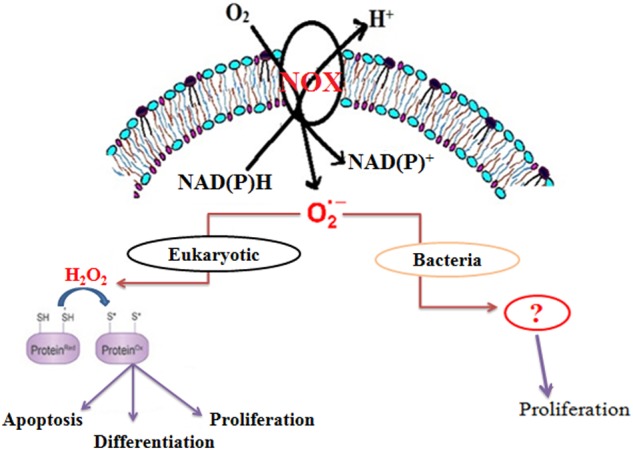
**Schematic representation of the role of endogenous superoxide in organisms**.

Although the role of ROS was earlier thought to be harmful, recent studies have highlighted them as significant physiological regulators of many cellular functions, such as transcriptional regulation, direct oxidative modification, protein turnover, protein-protein interaction, and enzyme modification (**Figure 1**) (Paulsen and Carroll, 2010; Finkel, 2011). ROS-mediated signaling is controlled by a delicate balance between its formation and its scavenging (Bailey-Serres and Mittler, 2006). Further experimental evidences suggest the involvement of ROS in the growth of higher eukaryotes (Nathan and Shiloh, 2000; Bloomfield and Pears, 2003; Foreman et al., 2003; Saran, 2003; Finkel, 2011), lower eukaryotes (Buetler et al., 2004), yeast, as well as some prokaryotes (Diaz et al., 2013). Among ROS, superoxide is the first oxygen radical to be generated in cells.

Membrane-bound NADPH oxidase is the major source of superoxide in eukaryotic cells (**Figure 1**) (Paulsen and Carroll, 2010). However, in bacteria, NADH oxidase has been reported to produce superoxide, which is further converted to either H_2_O_2_ or H_2_O (Nishiyama et al., 2001; Yang and Ma, 2007; Diaz et al., 2013). Recent studies also suggest that Mtb generates endogenous superoxide, which is involved in the critical management of the redox balance. Moreover, the increasing level of endogenous superoxide differentially affects the growth of *Mycobacterium* spp. (Tyagi et al., 2015). Interestingly, there is no report on the involvement of superoxide in the growth of *Mycobacterium* spp. In this study, we demonstrate that NADH oxidase-derived superoxide is involved in the growth *M. smegmatis*.

## Materials and Methods

### Chemicals, Media, and Strain

Dubos medium was purchased from DIFCO, USA and 2-hydroxyethidium was purchased from Noxygen, Germany. All other chemicals were purchased from Sigma-Aldrich, USA. *M. smegmatis* MC^2^155 strain was a gift from AstraZeneca, India. Sub-culturing of the strain was routinely performed on Dubos albumin agar slants. The stock was maintained at -70°C and sub-cultured once in liquid medium before inoculation in the experimental culture medium. *M. smegmatis* culture was inoculated in 20 mL Dubos broth in a 100-mL flask incubated at 37°C on an orbital shaker (Thermo Electron Model No.131 481; Thermo Electron Corp., Marietta, OH, USA) set at 150 rpm. Solutions of rotenone, antimycin A, DPI, menadione, pyrogallol, and dihydroxyethidum (DHE) were freshly prepared in dimethylsulfoxide (DMSO).

### Detection of Endogenous Superoxide Production in *M. smegmatis*

Superoxide production in *M. smegmatis* was detected by the following modified HPLC-based method, described earlier (Laurindo et al., 2008; Zielonka et al., 2008, 2009). Briefly, ∼2.2 × 10^8^ cells/mL of aerobically growing *M. smegmatis* culture was washed and re-suspended in 1 mL of *M. phlei* medium (Khan et al., 2008) containing diethylenetriaminepentaacetic acid (DTPA) and incubated with DHE at a final concentration of 50 μM at 37°C for 90 min. After incubation, the cell pellet was obtained by centrifugation at 10,000 rpm for 10 min at 4°C, washed twice with *M. phlei* medium, and re-suspended in 500 μL of the same medium containing 1–2% (v/v) of Triton X100. After mixing, an equal volume of acidified methanol [with 1% formic acid (v/v)] was added and the cell suspension mix was kept on ice for 90 min. The supernatant was collected after centrifugation at 15,000 rpm for 45 min at 4°C and filtered through a 0.2-μm membrane filter (Acrodisc 13 mm syringe filters, Pall Corporation). The filtrate was then analyzed using HPLC (Binary pump-1525, Fluorescence detector-2475, UV detector-2489, Sampler-2707, Waters, India). The chromatographic separation was performed on a C18 reverse phase column (Kinetex 5 μm C18 100A, 250 mm × 4.60 mm from Phenomenex, India). A gradient of solutions A [1% (v/v) formic acid in water] and B [1% (v/v) formic acid in acetonitrile] was used as the mobile phase at a flow rate of 0.4 mL/min. Chromatographic runs were started with 100% solution A, decreased linearly to 60% solution A during the first 8 min, and then kept constant at isocratic condition for a further 12 min. In the next 1 min, the gradient was set at 100% solution B, kept constant for a further 4 min, and equilibrated again with solution A for the next run. The 2-hydroxyethidium fluorescence was monitored by a fluorescence detector (excitation 480 nm, emission 580 nm). The area under the curve of 2-hydroxyethidium and ethidium were considered and their concentrations were calculated based on a standard plot.

Superoxide production in the crude membrane was assessed by the method described above. Briefly, 15 μg of the crude membrane protein was added to 500 μL of *M. phlei* medium containing 100 μM DTPA and 1 mM NADH. Freshly prepared DHE was added at a final concentration of 25 μM and the mix was incubated at 37°C, following which HPLC samples were prepared as described earlier in this section.

### Crude Membrane Preparation from *M. smegmatis*

Crude membrane was prepared by a previously described method (Drew et al., 2006) with some modifications, as follows: *M. smegmatis* cells were harvested by centrifuging at 2,000 × *g* for 15 min and re-suspended in 15 mL buffer containing 1X PBS (pH 8.0), 1 mg/mL EDTA, 0.002% lysozyme, and 1% protease inhibitor cocktail. The pellet was lysed by probe sonication (SONICS Vibra cell) of 10-s pulses for 20 min. The lysate was then subjected to centrifugation at 5,000 rpm for 20 min at 4°C to remove unbroken cells and cell debris. The supernatant was further subjected to ultracentrifugation at 120,000 × *g* for 90 min at 4°C (Sorvall WX Ultra Series, Thermo Electron Corporation) and the pellet containing membrane proteins was obtained after washing with cold PBS. This membrane pellet was finally re-suspended in 50 mM Tris-HCl buffer at pH 8.0

### Determination of NADH Oxidase Activity in Crude Membrane Preparation

NADH oxidase activity was measured in the crude membrane of *M. smegmatis* according to an assay described earlier (Reusch and Burger, 1974) with some modifications, as follows: 50 μg of membrane protein was added to 200 μL of 50 mM Tris buffer (pH 8) containing 100 μM dithiothreitol and 500 μM NADH in a 96-well quartz plate. The plate was incubated at 30°C with continuous shaking and the absorbance was monitored at 340 nm for 25 min (SpectraMax M5e Microplate Reader, Molecular Devices,USA). The inhibition of enzyme activity was determined by incubating various concentrations of DPI with above reaction mixture.

### Effect of ROS Modulators on the Growth of *M. smegmatis*

The growth of *M. smegmatis* was determined in the presence of different ROS modulators by following a previously described protocol (Khan et al., 2008). Briefly, 0.1% of mid-log phase culture, at OD_620_ of 1.0, was inoculated in 20 mL of Dubos medium at a final count of approximately 5.6 × 10^5^ cells/mL and incubated with different concentrations of ROS modulators for 4 days at 37°C with shaking at 120 rpm. *M. smegmatis* growth was estimated by determining the CFU/mL after 3 days of incubation on Dubos agar plate.

The effect of menadione and pyrogallol on bacillus growth was determined in the presence of DPI (15 μM). Mid-log phase cultures (2.2 × 10^8^ cells/mL) were supplemented with menadione (150 μM) and pyrogallol (300 μM) along with DPI and incubated for 24 h. Measurements of CFU/mL were determined after 3 days of incubation at 37°C on Dubos agar plate.

## Results

### Detection of Superoxide Generation in Aerobically Growing *M. smegmatis*

**FIGURE 2:**
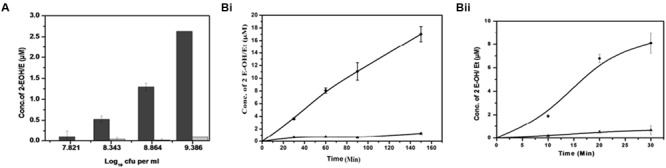
**Detection of superoxide produced by *M. smegmatis* cells. (A)** Cell concentration-dependent production of superoxide: Different concentrations of *M. smegmatis* cells were incubated with DHE for 90 min. After incubation, samples were further processed for HPLC analysis. Levels of 2-hydroxyethdium (2-EOH; dark bar) and ethidium (Et; gray bar) were determined from the standard plot.. The results represent the mean ± SD of three identical experiments. (*p* < 0.05). **(B)** Time-dependent increase in superoxide production in **(i)** *M. smegmatis* and **(ii)** *M. smegmatis* membrane preparation: Approximately 2.2 × 10^8^ cells/mL and 15 μg of the membrane protein were distinctly incubated with DHE at different time points. Samples were further processed for HPLC analysis. Levels of 2-EOH (•) and Et (▲) were determined from the standard plot. The results represent the mean ± SD of three identical experiments. (*p* < 0.05).

Superoxide is generated when one electron is added to an oxygen molecule and it plays an important role in the formation of other oxygen radicals (Turrens, 2003; Nicolopoulou-Stamati et al., 2004; Gülçin, 2006). Superoxide generation in *M. smegmatis* was measured using DHE (**Figure 2** and Supplementary Figure 1). DHE is a widely used probe that is oxidized by superoxide to yield fluorescent 2-hydroxyethidium and ethidium, both of which intercalate DNA and are efficiently retained by the cells (Fink et al., 2004; Laurindo et al., 2008; Zielonka et al., 2008, 2009). These two products, 2-hydroxyethidium and ethidium, were separated using HPLC at retention times of 18.6 and 19.2 min, respectively (Supplementary Figure 2). We found an increase in the 2-hydroxyethidium fluorescence, which was proportional to the increase in cell number (**Figure 2A**). A continuous production of 2-hydroxyethidium led to its steady accumulation over a period of 150 min in whole cells and 30 min in membrane preparation (**Figure 2B** and Supplementary Figure 2). However, the low level of ethidium was not significantly affected in these experiments (White et al., 2014).

### Determination of NADH Oxidase Activity in Crude Membrane Preparation

**FIGURE 3:**
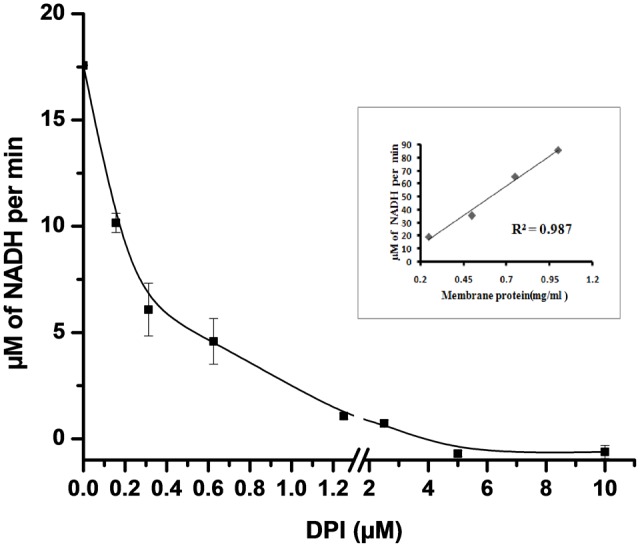
**Effect of DPI on NADH oxidase activity in crude membrane preparation.** 50 μg of crude membrane protein was incubated with different concentrations of DPI (0.15–10 μM) in 50 mM Tris buffer (pH 7.5) containing 500 μM of NADH for 25 min at 30°C. and inhibition activity determined measuring absorbance at 340 nm. *Inset*: Plot showing crude membrane protein vs. NADH conversion per min (R^2^ = 0.987). The results represent the mean ± SD of three identical experiments.

NADH oxidase generates superoxide by transferring electrons from NADH to molecular oxygen. NADH oxidase activity in crude membrane preparation was measured by the decrease in the absorbance of NADH at 340 nm for 25 min. We observed that the rate of NADH utilization linearly increased with the increase in the total crude membrane protein concentration (R^2^ = 0.987) in the reaction mixture (inset **Figure 3**). The rate of NADH utilization was found to decrease with increasing DPI concentrations which indicates potential inhibition of NADH oxidase activity (**Figure 3**).

### Identification of the Source/s of Superoxide Generation in *M. smegmatis*

**FIGURE 4:**
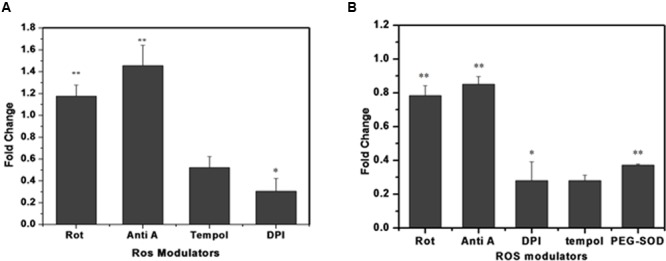
**Effect of different ROS modulators on superoxide production in *M. smegmatis.*** Rotenone (10 μM), antimycin A (10 μM), DPI (15 μM), tempol (10 μM), and PEG-SOD (5 units/mL) were incubated with **(A)** *M. smegmatis* and **(B)** membrane preparation. Samples were further processed for HPLC analysis. The results are expressed as fold change measured as the ratio of treated to untreated samples (control). The results represent the mean ± SD of three identical experiments. (^∗^*p* < 0.05; ^∗∗^*p* < 0.1).

To identify the possible source/s of superoxide generation in *M. smegmatis* cells, we tested the effects of various ROS modulators on *M. smegmatis* whole cells as well as on its crude membrane preparation and then monitored their effect on the oxidation of DHE to 2-hydroxyethidium (**Figure 4**) (Rottenberg et al., 2009). Tempol (4-hydroxy-Tempo) a cell permeable nitroxide of the superoxide dismutase (SOD) mimic class inhibited the production of 2-hydroxyethidium substantially by 0.52 ± 0.084 and 0.28 ± 0.032 fold in whole cells and in the membrane preparation, respectively, compared to the untreated control normalized value of 1 (**Figures 4A,B**) (Nandakumar et al., 2014) Polyethylene glycol-SOD (PEG-SOD) also inhibited 2-hydroxyethidium production by 0.37 ± 0.011 fold in the crude membrane preparation compared to the untreated control. DPI reduced the maximum of 2-hydroxyethidium production by 0.30 ± 0.093 and 0.27 ± 0.102 fold in whole cells and in the membrane preparation, respectively. The electron transport chain (ETC) inhibitors, rotenone (complex I, NADH dehydrogenase) and antimycin A (complex III, cytochrome-bc1 complex), did not inhibit the production of 2-hydroxyethidium; instead, resulted in a 1.17 ± 0.12 and 1.45 ± 0.12 fold increase in 2-hydroxyethidium levels, respectively, in whole cells, as reported earlier (Verkaart et al., 2007; Piskernik et al., 2008). Similarly, in membrane preparations, rotenone and antimycin A inhibited 2-hydroxyethidium production only by 0.78 ± 0.068 and 0.85 ± 0.046 fold, respectively (**Figure 4**). Interestingly, *M. smegmatis* cells were viable after treatment with these ROS modulators for 90 min, indicating that the observed fold changes in 2-hydroxyethidum were not due to cell death (data not shown).

### Effect of ROS Modulators on the Growth of *M. smegmatis*

**FIGURE 5:**
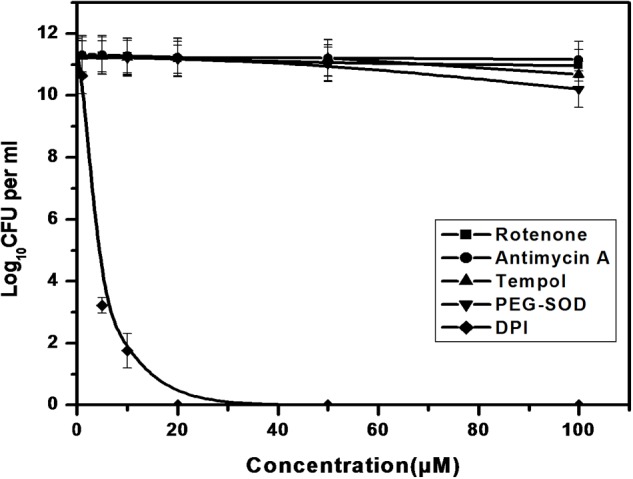
**Effect of ROS modulators on the growth of *M. smegmatis*.** Log phase culture of *M. smegmatis* consisting of ∼5.6 × 10^5^ cells/mL was grown in the presence of the ROS modulators for 4 days, following which the number of CFUs was calculated. Data shown are representative of four independent experiments.

We further investigated the possible adverse effects of ROS modulators on *M. smegmatis* growth. ETC inhibitors (Rotenone and Antimycin A), tempol and PEG-SOD did not affect the CFU counts of *M. smegmatis* even 10-fold higher concentration used than the superoxide detection experiment (**Figure 5**). In contrast, only DPI inhibited the growth of *M. smegmatis*, possibly owing to the depletion of superoxide within the cell (**Figure 5**).

### Superoxide Involvement in *M. smegmatis* Growth

**FIGURE 6:**
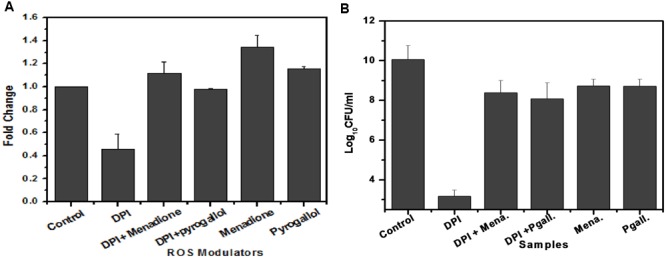
**Involvement of superoxide in the growth of *M. smegmatis*. (A)** Effect of ROS modulators and generators on superoxide production by *M. smegmatis. M. smegmatis* cells were incubated with DPI (15 μM), DPI (15 μM) + menadione (150 μM), DPI (15 μM) + pyrogallol (300 μM), menadione (150 μM), and pyrogallol (300 μM) for 90 min. Samples were further processed as described in **Figure 3**. The results represent the mean ± SD of three identical experiments. (*p* < 0.05). **(B)** Effect of ROS modulators and generators on the growth of *M. smegmatis*. Log phase culture of *M. smegmatis* consisting of ∼2.3 × 10^8^ cells/mL was incubated with DPI (15 μM), DPI (15 μM) + menadione (150 μM), DPI (15 μM) + pyrogallol (300 μM), menadione (150 μM), and pyrogallol (300 μM) for 24 h, after which plating was done on Dubos agar plates. CFU measurements were performed after 3 days of incubation. Data shown are representative of three independent experiments.

To determine the involvement of superoxide in *M. smegmatis* growth, we added menadione and pyrogallol, separately and along with DPI, to the *M. smegmatis* culture and observed their effect on superoxide production as well as bacilli growth. Menadione (150 μM) and pyrogallol (300 μM) increased superoxide production by 1.41 ± 0.062 and 1.14 ± 0.041 fold, respectively, when added separately, and by 0.45 ± 0.13 to 1.12 ± 0.101 fold and 0.99 ± 0.022 fold, respectively, when added along with DPI (**Figure 6A**). DPI reduced the bacilli growth by a difference of 6.8 log_10_ compared to the growth in the untreated control. This effect was reversed when menadione or pyrogallol was added, showing a difference of only 1.5 or 1.9 log_10_, respectively, compared to the growth in the untreated control (**Figure 6B**). To optimize the concentration of menadione and pyrogallol, a prior dose response study was carried out in the presence of a fixed concentration of DPI (data not shown). Thus, this study establishes that DPI inhibits growth through the inhibition of superoxide production in *M. smegmatis*.

## Discussion

Earlier studies have demonstrated that the level of superoxide production is proportional to the availability of O_2_ in the environment (Bloomfield and Pears, 2003; Foreman et al., 2003; Buetler et al., 2004; Shi et al., 2008). Our initial studies using DHE have clearly established that actively growing *M. smegmatis* bacilli continuously produce superoxide radicals (**Figure 2**). The superoxide level needs a critical management by mycobacterial antioxidant enzymes to ensure its beneficial functioning in the bacilli. Thus, this justifies the expression of antioxidant enzymes in axenic cultures as reported earlier (Muller et al., 2004; Suresh et al., 2010).

Previous studies on different prokaryotic organisms have shown that NADH oxidase and menaquinone, associated with ETC, are the two major sites of superoxide generation in these organisms (Chandel et al., 2000; Zalba et al., 2000; Hasan et al., 2006). DPI inhibits NADPH oxidase in eukaryotes and has a homologous mechanism similar to NADH oxidase present in bacteria (Li and Trush, 1998; Korshunov and Imlay, 2006; Diaz et al., 2013). Additionally, inhibition of NADH oxidation by DPI in the crude membrane preparation confirms the presence of NADH oxidase-like enzyme in the membrane of *M. smegmatis* (**Figure 3**).

In accordance with earlier studies, our results clearly indicate that DPI significantly inhibits superoxide production in whole cells as well as in the membrane preparation of *M. smegmatis* (**Figure 4**) However, contribution of ETC in superoxide production remains unclear due to the lack of significant effect of any of the ETC inhibitors in *M. smegmatis* Thus, we identified NADH oxidase as the most probable source of superoxide production in *M. smegmatis*. Interestingly *M. smegmatis* possesses four isozymes of NADH oxidase (KEGG database). Further studies on these NADH oxidase isozymes will clarify their role in superoxide production.

In our study, DPI was found to decrease the superoxide generation as well as the growth of the *M. smegmatis* bacilli (**Figure 6**). Reversion of the effect of DPI on the bacilli growth by superoxide generators (menadione and pyrogallol) confirmed the involvement of superoxide in *M. smegmatis* growth (**Figure 6B**). Previous *in vivo* studies have shown similar inhibitory effects of plant-derived antioxidants against *M. intracellulare* as well as against *M. abscessus* (Reddy et al., 2007). As reported earlier, rotenone and antimycin A neither inhibited superoxide production (Verkaart et al., 2007; Piskernik et al., 2008) nor showed any effect on the bacilli growth (**Figure 5**) (Parrish et al., 2004; Megehee et al., 2006). PEG-SOD depleted superoxide in membrane preparations, but its inefficiency to inhibit the bacilli growth could be due to a poor penetration through the mycobacterial cell membrane, which could be attributed to its high molecular weight. In contrast to DPI, tempol did not inhibit superoxide production. Tempol has been reported to facilitate the dismutation of superoxide like SOD, to hydrogen peroxide and to exhibit a catalase-like activity (Wilcox and Pearlman, 2008). Therefore, the inability of tempol to inhibit growth, albeit with the reduction in the superoxide level, could be attributed to the possible involvement of other ROS. In the case of DPI, inhibition of the superoxide source could possibly lead to a significant decrease in the subsequent generation of other ROS. Under physiological conditions, the role of superoxide will remain important, as it is also the primary source of other oxygen radicals. Our results suggest that other ROS could be possibly associated with the growth of the cells. Further studies will be necessary to find out which ROS species is more closely involved in the regulation of mycobacterial growth.

The involvement of superoxide in the regulation of mycobacterial growth is a new finding and is in accordance with earlier reports on the involvement of superoxide in the growth regulation of some prokaryotes and eukaryotes (Bloomfield and Pears, 2003; Foreman et al., 2003; Buetler et al., 2004; Diaz et al., 2013). Thus, our study provides new insights into mycobacterial growth regulation during hypoxia and under vitamin C-induced dormancy as well (Wayne and Hayes, 1996; Taneja et al., 2010). In summary, this study demonstrates that a significant amount of superoxide is actively generated by *M. smegmatis* and that NADH oxidase is its major contributor. Our results also suggest that superoxide may play an important role in the growth regulation of the *M. smegmatis* bacilli.

## Author Contributions

This study was designed and done by all authors. AY and KS participated equally in all this work, whereas MA helped them with membrane preparation and related studies under the guidance of DS.

## Conflict of Interest Statement

The authors declare that the research was conducted in the absence of any commercial or financial relationships that could be construed as a potential conflict of interest.
