# Supplementary material for: Superoxide Generation and Its Involvement in the Growth of Mycobacterium smegmatis
Source: Front Microbiol. 2017 Jan 30;8:105. doi: 10.3389/fmicb.2017.00105 (PMC5276846; doi:10.3389/fmicb.2017.00105)

Supplementary Material

Figure 1

Detection of endogenous superoxide production by *Mycobacterium smegmatis* cells using fluorescent microscopy

M. smegmatis mid log phase culture of 0.65 OD_{620} was washed with PBS and re-suspended in PBS containing 10µM DHE. These cells were incubated for two hours at 37°C on an orbital shaker at 150 rpm. Cell were washed with PBS and visualized by fluorescent microscopy (Leitz Wetzlar, Germany) with I2 excitation filter (530nm) and Emission filter (600nm) under 400X magnification.

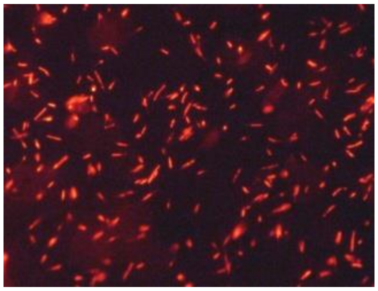

Figure 2

HPLC chromatograms showing time dependent increase in 2-hydroxyethidium peak

This is the supplementary figure for Fig. 1B in the main text. The chromatograms taken at respective time points as 10 minutes (red), 20 minutes (blue), 30 minutes (green), 60 minutes (cyan) to 120 minutes (black) after DHE incubation as described in "Materials and Methods". (Peaks were merged by Breeze® Waters HPLC software)

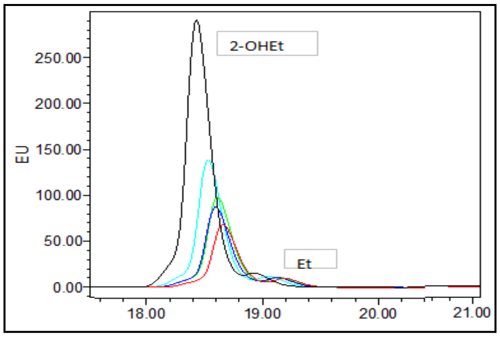

Supplement: Supplementary file 1 [file Image_1.PDF]
